# Multi-field coupled dynamics for a movable tooth drive system integrated with shape memory alloys

**DOI:** 10.1016/j.heliyon.2023.e17531

**Published:** 2023-06-26

**Authors:** Lizhong Xu, Zhenglong Fu

**Affiliations:** Mechanical Engineering Institute, Yanshan University, Qinhuangdao, 066004, China

**Keywords:** Multi-field coupled, Dynamics, Shape memory alloys, Movable tooth drive, Integrated

## Abstract

The harmonic movable tooth drive system integrated with shape memory alloys has a small size and a large output torque. Its dynamics performance is the key factor for evaluating the drive system. Here, for the drive system, based on its structure and working principle, the coupled dynamics equations are deduced. Using the equations, changes of the natural frequencies of the drive system during the operation are investigated. Effects of the system parameters and SMA wires phase change process on the natural frequencies are analyzed. The nonlinear resonant frequencies of the drive system and its amplitude-frequency relationship are studied. Results show that natural frequencies of the drive system change periodically which is caused by SMA phase transformation during operation. The eccentricity, movable tooth radius, the wave generator radius and SMA wire length have also important effects on the natural frequencies of the drive system. The nonlinear resonant frequencies are smaller than linear resonant frequencies. In the design of the drive system, the coupled nonlinear effects of the temperature, phase change, stress and strain of the SMA wires, and the system parameters of the movable tooth drive system should be considered. In this paper, the coupled nonlinear dynamics model of the harmonic movable tooth drive system integrated with shape memory alloys is proposed in which the coupled effects of the temperature, phase change, stress and strain of the SMA, and the system parameters of the movable tooth drive system are considered.

## 1 Introduction

Shape memory alloy (SMA) has the advantages of high power density, high recovery strain, clean and silent, low working voltage and simple structure [1,2]. These advantages make SMA very suitable for low-speed, compact, large-load actuators. Actuators based on shape memory effect have been widely used in aerospace, robotics, biomedical engineering and automotive engineering [[3], [4], [5]].

Bunton used a SMA wire spring to create a model of a human eyeball that can rotate from side to side [6]. Tadesse embedded many SMA filaments into the robot face and controlled the deformation of SMA filaments to realize the movement similar to the contraction of human facial muscles [7]. Gaissert designed a biomimetic dragonfly with stable flight in which SMA was embedded in its head, tail and other joint structures, and arbitrary flight posture could be realized by controlling SMA [8]. Kim designed an SMA-driven biomimetic earthworm with an overall size of 300 mm and a maximum crawling speed of about 10 mm/min was obtained [9].

SMA actuators include two types: linear motion actuators and rotary motion actuators. Linear motion actuators are simple because they work directly using the linear shrinkage deformation of SMA. The rotating actuator needs to transform the linear shrinkage deformation of SMA into rotational motion.

Tanaka proposed a SMA rotation driver in which two SMA wires with pulleys were placed opposite each other and the central axis could be rotated over a limited range of angles [10]. Song winded the SMA wire on a team of the pulleys, thus the effective length of SMA wire was increased and the rotation angle range of the SMA actuator was enlarged [11]. Based on the torsional SMA rod, Tobushi proposed a miniature rotary actuator [12]. Yuan investigated a rotary actuator driven by helical SMA wire and achieved a fully reversible rotation of 150° [13]. Manfredi investigated a lightweight, low-power miniature rotary actuator with on-board contactless position and force sensors, where the generated torque was controlled by on-board force sensors, and the energy consumption was experimentally investigated [14]. Doroudchi investigated a fast response SMA-driven rotary actuator in which a pair of inversely configured SMA wires was used to increase drive speed and achieve bidirectional rotation without the use of springs [15]. Takeda studied a reciprocating rotary actuator of SMA tapes in which according to the torsional deformation characteristics of SMA tapes, a graphical design method of the actuator was proposed [16]. Moghadam investigated a position tracking control method of SMA driven rotating actuator using torque and position measurement, and evaluated the performance and accuracy of the controller by experiments [17]. Moghadam proposed a fast response control method of SMA driven rotating actuator based on robust sliding mode control, which uses both SMA strain and temperature as feedback signals [18]. Jang studied a rotating actuator in which the SMA wire was wound, folded and integrated into the rotating actuator, so that it could rotate in the opposite direction simply by changing the torsion direction [19].

The structure of above-mentioned SMA actuators is simple, but they are unable to achieve a full cycle of continuous rotation. Yan used five SMA wires to drive one shaft to rotate continuously in one direction, and its output speed is 0.28 r/min [20,21]. Hwang combined SMA drive principle with the step motor technique to propose a novel continuous rotation SMA motor, in which the gears were used and the meshing forces were used to realize relatively large torque output [[22], [23], [24]]. Xu proposed a harmonic movable tooth drive system integrated with shape memory alloys and investigated the multi-field coupled static forces and the output torque of the drive system under pulsed current [25].

The harmonic movable tooth drive system integrated with SMA uses the simplest way to generate traveling waves and only two SMA wires are used to drive movable tooth drive system for continuous rotation. It has a small size and a large output torque. So, it has a wide application prospect. Dynamics performance is the key factor of the performance design for the drive system and other physical phenomenon [26]. However, dynamics performance about the drive system has not been investigated yet.

In this paper, for the harmonic movable tooth drive system integrated with SMA, its structure and working principle are introduced. The coupled dynamics model of the drive system is proposed and the coupled dynamics equations are deduced, in which temperature, phase change, stress and strain of the SMA wires, and the system parameters of the movable tooth drive system are considered simultaneously. Using the equations, changes of the natural frequencies of the drive system during the operation are investigated. Effects of the system parameters and SMA wires phase change process on the natural frequencies of the drive system are analyzed. The nonlinear resonant frequencies of the drive system and its amplitude-frequency relationship are studied. Changes of the natural frequencies along with the main parameters are analyzed for the five typical vibration modes. Results show that natural frequencies of the drive system change periodically which is caused by SMA phase transformation during operation. The eccentricity, movable tooth radius, the wave generator radius and SMA wire length have also significant effects on the natural frequencies of the drive system. The nonlinear resonant frequencies are smaller than linear resonant frequencies. With increase of the order number, the relative error between the nonlinear and linear resonant frequencies decreases.

## 2 Structure and operation principle

**Fig. 1:**
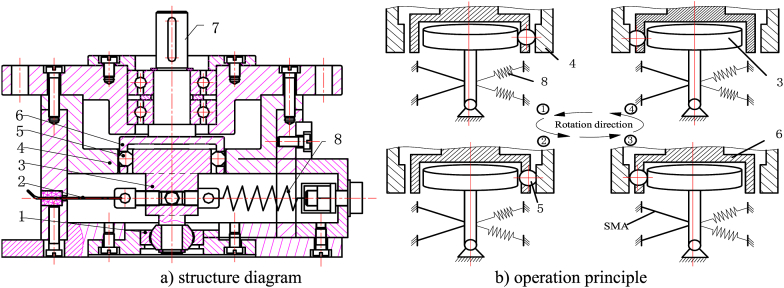
Structure diagram and operation principle of integrated SMA movable tooth drive system.

The structure diagram of electromechanical integrated SMA movable tooth drive system is shown in Fig. 1. Here, 1 is a rolling bearing, 2 is SMA wire, 3 is swing rod, 4 is central ring, 5 is movable tooth, 6 is wave generator, 7 is output shaft, and 8 is bias spring (see Fig. 1a). The system is composed of two parts: transmission and drive. The SMA wire drive and movable tooth transmission are integrated together by swing rod. The driving part is composed of two SMA wires, which are uniformly distributed on the same plane. Under the pre-stress of the bias spring, the SMA wires stretch and deform, and the twinned martensite is transformed to non-twinned martensite.

When one SMA wire is heated by the current, the SMA wire will change from non-twin martensite to austenite when the temperature reaches a certain temperature, which causes the SMA wire to shrink and pulls the swing rod to one side of the SMA wire. When the SMA wire is cooled by turning off the current, the SMA wire will transition from austenite to non-twinned martensite, the SMA will return to its original state, and the swing rod will return by the pre-stress of the bias spring. By heating and cooling the two SMA wires, the swing rod can move continuously. When the two SMA wires are coordinated, the swing rod can move in the SMA wire plane with traveling wave trajectory. By the amplification of the swing rod, the wave generator is driven to realize the traveling wave movement in the movable tooth transmission plane, so that the torque is output through the movable tooth transmission (see Fig. 1b).

## 3 Coupled dynamics equations

**Fig. 2:**
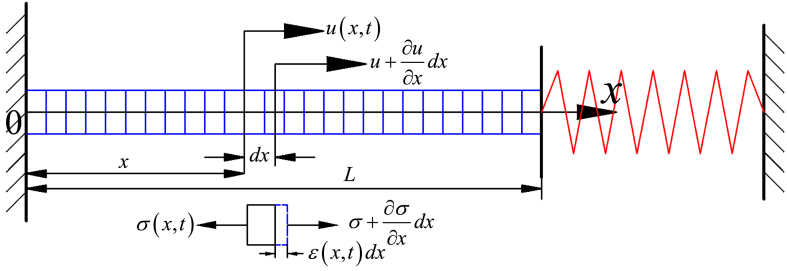
Longitudinal vibration model of the X-channel SMA wire.

X and Y represent two channels of SMA wires. Firstly, the dynamics model of X channel of SMA wire is established. The longitudinal vibration model of the X-channel SMA wire is shown in Fig. 2. Here, *u*(x,t) represents the displacement of SMA wire at time *t* at the x-section, σ(x,t) represents the horizontal stress on SMA wire, and ε(x,t) represents the axial strain.

From Fig. 2, it is known that at coordinate *x*, using Newton's second law, the dynamics equation of the X-channel SMA wire can be given by [27].

(1) $$E_{M}\frac{\partial^{2}u}{\partial x^{2}}=\rho \frac{\partial^{2}u}{\partial t^{2}}$$

Letting *u*(x,t) = *X*(x)*F*(t) and substituting it into (1), yields

(2) $$\frac{d^{2}F\left(t\right)}{dt^{2}}+\omega_{j}^{2}F\left(t\right)=0$$

(3) $$\frac{d^{2}X\left(x\right)}{dx^{2}}+\frac{\omega_{j}^{2}}{a^{2}}X\left(x\right)=0$$

where *a*=(E/ρ)^0.5^.

In a same manner, the dynamics equation of the Y-channel SMA wire can also be given. From Eqs.((2) and (3), the mode functions of the two channel SMA wires can be given by

(4) $$\left\{ \begin{array}{l} X\left(x\right)=B_{1}\;\sin\frac{\omega_{j}}{a}x+C_{1}\;\cos\frac{\omega_{j}}{a}x\\ Y\left(y\right)=B_{1}\;\sin\frac{\omega_{j}}{a}y+C_{1}\;\cos\frac{\omega_{j}}{a}y \end{array}\right.$$

where $\omega_{j}$ is the natural frequency of the two channel SMA wires.

**Fig. 3:**
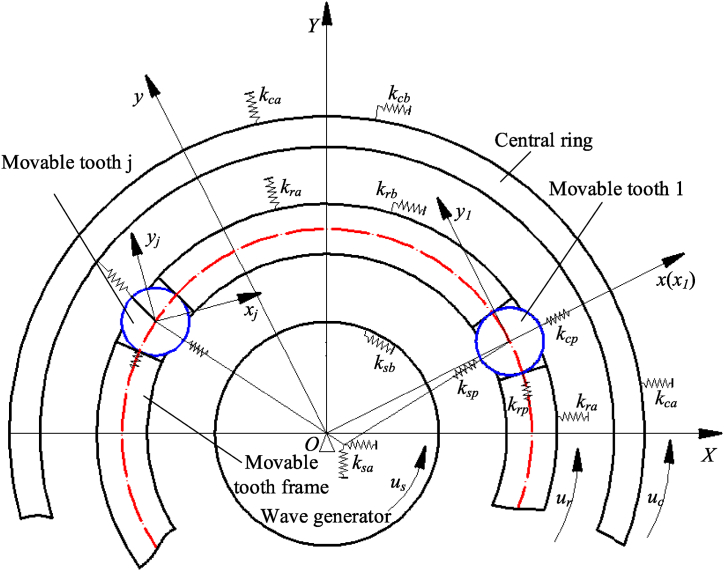
Dynamic model of SMA movable tooth transmission system.

Fig. 3 shows the dynamic model of the SMA movable tooth transmission system. Here, XOY is the fixed coordinate system of the movable tooth transmission system, xoy is the coordinate system of the movable tooth frame, which is fixed to the movable tooth frame and rotates with the movable tooth frame. Its x-axis passes through the theoretical center of the first movable tooth. x_i_oy_i_ is the coordinate system of the ith movable tooth, and its origin is located at the theoretical center of the ith movable tooth. The two axes of the movable tooth coordinate system are always parallel to the two axes of the coordinate system xoy.

The displacement vector of each component and the corresponding mass matrix are defined as

(5) $$\left\{ \begin{array}{c} q_{j}={\left[x_{j}\;y_{j}\;u_{j}\right]}^{T}\\ M_{j}=diag\left[m_{j}\;m_{j}\;I_{j}/{r_{j}}^{2}\right] \end{array}\right.$$

where $x_{j}$ and $y_{j}$ are the vibration displacements of the element $j\;\left(j=r,s,c,p_{1},\cdots p_{Z}\right)$, r represents movable tooth frame, s wave generator, c central ring, p movable tooth, z is the movable tooth number in mesh; $u_{j}$ is the linear displacement of the element j in circumferential direction, $u_{j}=r_{j}\theta_{j}$, $r_{j}$ is the radius of the element j, $\theta_{j}$ is the angular displacement of the element $j\;\left(j=r,s,c,p_{1},\cdots p_{Z}\right)$, $m_{j}$ is the mass of the element j, and $I_{j}$ is the moment of inertia of the element j.

**Fig. 4:**
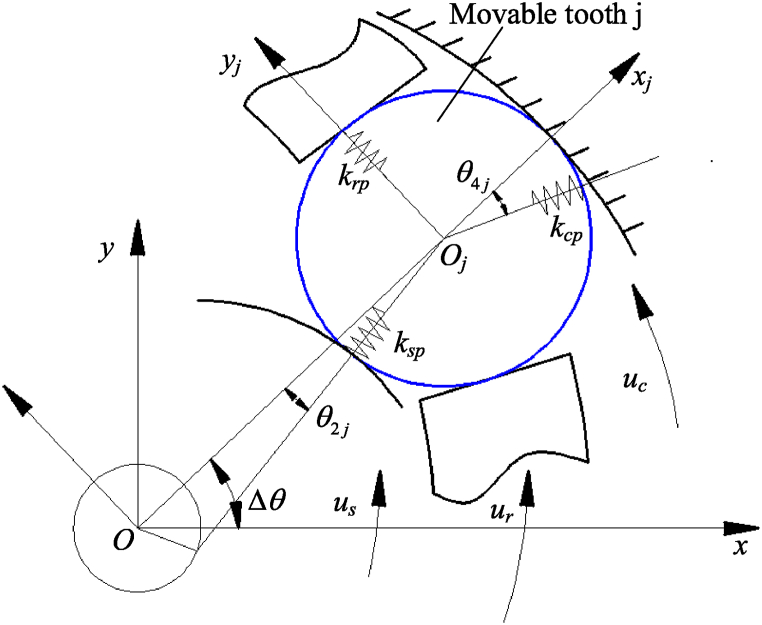
Relative positions of the movable tooth j and other components.

At time *t*, the relative positions of the movable tooth j and other components are shown in Fig. 4. Assuming that the contact mode between the movable tooth j and other components is linear elastic deformation, k_sp_, k_rp_, and k_cp_ respectively represent the meshing stiffness between the movable tooth j and wave generator, movable tooth frame, and central wheel.

δ_sj_ represents the relative displacement between the wave generator and the movable tooth j. δ_cj_ represents the relative displacement between the central wheel and the movable tooth j. δ_rj_ represents the relative displacement between the movable tooth frame and the movable tooth j. It can be given from Fig. 4.

(6) $$\delta_{sj}=\left(x_{s}-x_{j}\right)\cos\left(\Delta \theta -\theta_{2j}\right)+\left(y_{s}-y_{j}\right)\sin\left(\Delta \theta -\theta_{2j}\right)-u_{s}\;\sin\;\theta_{2j}$$

(7) $$\delta_{cj}=\left(x_{c}-x_{j}\right)\cos\left(\Delta \theta +\theta_{4j}\right)+\left(y_{c}-y_{j}\right)\sin\left(\Delta \theta +\theta_{4j}\right)+u_{r}\;\sin\;\theta_{4j}$$

(8) $$\delta_{rj}=\left(x_{r}-x_{j}\right)\cos\left(\Delta \theta +\pi /2\right)+\left(y_{s}-y_{j}\right)\sin\left(\Delta \theta +\pi /2\right)+u_{s}$$

whereΔθ is the position angle of the movable tooth j, θ_2j_ is the angle between common normal of the wave generator and the movable tooth j and negative x axis, θ_4j_ is the angle between common normal of the central wheel and the movable tooth j and positive x axis.

Considering the elastic contact between the wave generator and the movable tooth, and the fixed connection of the SMA wire and bias spring, the elastic dynamic differential equation of the wave generator can be given as below

(9) $$\left\{ \begin{array}{l} m_{s}\overset{..}{x_{s}}+\sum_{j=1}^{Z}\left(k_{sp}\delta_{sj}\;\cos\left(\Delta \theta -\theta_{2j}\right)\right)+k_{sa}x_{s}+\left(\frac{AE_{1}}{L_{0}}-K_{sp}\right)x_{s}\;\cos\;\theta_{r}+\left(\frac{AE_{2}}{L_{0}}-K_{sp}\right)x_{s}\;\sin\;\theta_{r}=0\\ m_{s}\overset{..}{y_{s}}+\sum_{j=1}^{Z}\left(k_{sp}\delta_{sj}\;\sin\left(\Delta \theta -\theta_{2j}\right)\right)+k_{sa}y_{s}+\left(\frac{AE_{2}}{L_{0}}-K_{sp}\right)y_{s}\;\cos\;\theta_{r}-\left(\frac{AE_{1}}{L_{0}}-K_{sp}\right)y_{s}\;\sin\;\theta_{r}=0\\ \left(I_{s}/{r_{s}}^{2}\right)\overset{..}{u_{s}}-\sum_{j=1}^{Z}\left(k_{sp}\delta_{sj}\;\sin\;\theta_{2j}\right)+k_{sb}u_{s}=0 \end{array}\right.$$

where *k*_sa_ is the radial support stiffness of wave generator, *k*_sb_ is the tangential torsional stiffness of wave generator.

Substituting Eqs. (5), (6), (7), (8), (9), the elastic dynamic differential equation of the wave generator can be written in matrix form as below

(10) $$M_{s}\overset{..}{q_{s}}+\left(K_{bs}+\sum_{j=1}^{Z}K_{sj}\right)q_{s}+\sum_{i=1}^{Z}\left(K_{spj}q_{j}\right)=0$$

where $M_{s}=diag\left[m_{s}\;m_{s}\;I_{s}/r_{s}^{2}\right]$, $K_{bs}=diag\left[k_{sa}\;k_{sa}\;k_{sb}\right]$,

$$n_{1j}=\cos\left(\Delta \theta -\theta_{2j}\right),n_{2j}=\sin\left(\Delta \theta -\theta_{2j}\right),n_{3j}=\sin\;\theta_{2j}$$

$$K_{sj}=k_{sp}\left[\begin{array}{ccc} n_{1j}^{2} & n_{1j}n_{2j} & -n_{1j}n_{3j}\\ n_{1j}n_{2j} & n_{2j}^{2} & -n_{2j}n_{3j}\\ -n_{1j}n_{3j} & -n_{2j}n_{3j} & n_{3j}^{2} \end{array}\right],K_{spj}=k_{sp}\left[\begin{array}{ccc} -n_{1j}^{2} & -n_{1j}n_{2j} & 0\\ -n_{1j}n_{2j} & -n_{2j}^{2} & 0\\ n_{1j}n_{3j} & n_{2j}n_{3j} & 0 \end{array}\right]$$

The central wheel contacts to the movable tooth, and is fixed to the base. Its dynamics equation can be given by

(11) $$\left\{ \begin{array}{c} m_{c}\overset{..}{x_{c}}+\sum_{j=1}^{Z}\left(k_{cp}\delta_{cj}\;\cos\left(\Delta \theta +\theta_{4j}\right)\right)+k_{ca}x_{c}=0\\ m_{c}\overset{..}{y_{c}}+\sum_{j=1}^{Z}\left(k_{cp}\delta_{cj}\;\sin\left(\Delta \theta +\theta_{4j}\right)\right)+k_{ca}y_{c}=0\\ \left(I_{c}/{r_{c}}^{2}\right)\overset{..}{u_{c}}-\sum_{j=1}^{Z}\left(k_{cp}\delta_{cj}\;\sin\;\varphi_{4j}\right)+k_{cb}u_{c}=0 \end{array}\right.$$

where *k*_ca_ is the radial support stiffness of central wheel, *k*_cb_ is the tangential torsional stiffness of central wheel.

Eq. (11) can be written in matrix form as below

(12) $$M_{c}{\ddot{q}}_{c}+\left(K_{bc}+\sum_{j=1}^{Z}K_{cj}\right)q_{c}+\sum_{j=1}^{Z}\left(K_{cpj}q_{j}\right)=0$$

where $M_{c}=diag\left[m_{c}\;m_{c}\;I_{c}/r_{c}^{2}\right]，K_{bc}=diag\left[k_{ca}\;k_{ca}\;k_{cb}\right]$.

$$n_{4j}=\cos\left(\Delta \theta +\theta_{4j}\right)，n_{5j}=\sin\left(\Delta \theta +\theta_{4j}\right)，n_{6j}=\sin\;\theta_{4j}$$

$$K_{cj}=k_{cp}\left[\begin{array}{ccc} n_{4j}^{2} & n_{4j}n_{5j} & n_{4j}n_{6j}\\ n_{4j}n_{5j} & n_{5j}^{2} & n_{5j}n_{6j}\\ n_{4j}n_{6j} & n_{5j}n_{6j} & n_{6j}^{2} \end{array}\right]，K_{cpj}=k_{cp}\left[\begin{array}{ccc} -n_{4j}^{2} & -n_{4j}n_{5j} & 0\\ -n_{4j}n_{5j} & -n_{5j}^{2} & 0\\ -n_{4j}n_{6j} & -n_{5j}n_{6j} & 0 \end{array}\right]$$

in a same manner, the dynamics equation of the movable tooth frame is

(13) $$\left\{ \begin{array}{c} m_{r}\overset{..}{x_{r}}+\sum_{j=1}^{Z}\left(k_{rp}\delta_{rj}\;\cos\left(\Delta \theta +\pi /2\right)\right)+k_{ra}x_{r}=0\\ m_{r}\overset{..}{y_{r}}+\sum_{j=1}^{Z}\left(k_{rp}\delta_{rj}\;\sin\left(\Delta \theta +\pi /2\right)\right)+k_{ra}y_{r}=0\\ \left(I_{r}/{r_{r}}^{2}\right)\overset{..}{u_{r}}+\sum_{j=1}^{Z}\left(k_{rp}\delta_{rj}\right)+k_{rb}u_{r}=0 \end{array}\right.$$

where *k*_ra_ is the radial support stiffness of movable tooth frame, *k*_rb_ is the tangential torsional stiffness of movable tooth frame.

Eq. (13) can be written in matrix form as below

(14) $$M_{r}\overset{..}{q_{r}}+\left(K_{br}+\sum_{j=1}^{z}K_{rj}\right)q_{r}+\sum_{j=1}^{z}\left(K_{rpj}q_{j}\right)=0$$

where $M_{r}=diag\left[m_{r}\;m_{r}\;I_{r}/r_{r}^{2}\right]$， $K_{br}=diag\left[k_{ra}\;k_{ra}\;k_{rb}\right]$，

$$n_{7j}=\cos\left(\Delta \theta +\pi /2\right)，\;n_{8j}=\sin\left(\Delta \theta +\pi /2\right)$$

$$K_{rj}=k_{rp}\left[\begin{array}{ccc} n_{7j}^{2} & n_{7j}n_{8j} & n_{7j}\\ n_{7j}n_{8j} & n_{8j}^{2} & n_{8j}\\ n_{7j} & n_{8j} & 1 \end{array}\right]，K_{rpj}=k_{rp}\left[\begin{array}{ccc} -n_{7j}^{2} & -n_{7j}n_{8j} & 0\\ -n_{7j}n_{8j} & -n_{8j}^{2} & 0\\ -n_{7j} & -n_{8i} & 0 \end{array}\right]$$

in a same manner, the dynamics equation of the movable tooth j is

(15) $$\left\{ \begin{array}{c} m_{p}\overset{..}{x_{j}}-k_{sp}\delta_{sj}\;\cos\left(\Delta \theta -\theta_{2j}\right)-k_{cp}\delta_{cj}\;\cos\left(\Delta \theta +\theta_{4j}\right)-k_{rp}\delta_{rj}\;\cos\left(\Delta \theta +\pi /2\right)=0\\ m_{p}\overset{..}{y_{j}}-k_{sp}\delta_{sj}\;\sin\left(\Delta \theta -\theta_{2j}\right)-k_{cp}\delta_{cj}\;\sin\left(\Delta \theta +\theta_{4j}\right)-k_{rp}\delta_{rj}\;\sin\left(\Delta \theta +\pi /2\right)=0\\ \left(I_{p}/{r_{p}}^{2}\right)\overset{..}{u_{j}}=0 \end{array}\right.$$

Eq. (15) can be written in matrix form as below

(16) $$M_{p}\overset{..}{q_{j}}+\left(K_{s1}^{j}+K_{c1}^{j}+K_{r1}^{j}\right)q_{j}+K_{psj}q_{s}+K_{pcj}q_{c}+K_{prj}q_{r}=0$$

where $M_{p}=diag\left[m_{p}\;m_{p}\;I_{p}/r_{p}^{2}\right]$， $K_{s1}^{j}=k_{sp}\left[\begin{array}{ccc} n_{1j}^{2} & n_{1j}n_{2j} & 0\\ n_{1j}n_{2j} & n_{2j}^{2} & 0\\ 0 & 0 & 0 \end{array}\right]$,

$$K_{psj}=k_{sp}\left[\begin{array}{ccc} -n_{1j}^{2} & -n_{1j}n_{2j} & n_{1j}n_{3j}\\ -n_{1j}n_{2j} & -n_{2j}^{2} & n_{2j}n_{3j}\\ 0 & 0 & 0 \end{array}\right]={\left(K_{spj}\right)}^{T}，K_{c1}^{j}=k_{cp}\left[\begin{array}{ccc} n_{4j}^{2} & n_{4j}n_{5j} & 0\\ n_{4j}n_{5j} & n_{5j}^{2} & 0\\ 0 & 0 & 0 \end{array}\right]，\;K_{pcj}=k_{cp}\left[\begin{array}{ccc} -n_{4j}^{2} & -n_{4j}n_{5j} & -n_{4j}n_{6j}\\ -n_{4j}n_{5j} & -n_{5j}^{2} & -n_{5j}n_{6j}\\ 0 & 0 & 0 \end{array}\right]={\left(K_{cpj}\right)}^{T}，K_{r1}^{j}=k_{rp}\left[\begin{array}{ccc} n_{7j}^{2} & n_{7j}n_{8j} & 0\\ n_{7j}n_{8j} & n_{7j}^{2} & 0\\ 0 & 0 & 0 \end{array}\right]，K_{prj}=k_{rp}\left[\begin{array}{ccc} -n_{7j}^{2} & -n_{7j}n_{8j} & 0\\ -n_{7j}n_{8j} & -n_{8j}^{2} & 0\\ 0 & 0 & 0 \end{array}\right]={\left(K_{rpj}\right)}^{T}$$

Combining Eqs. (10), (12), (14) with (16), and considering damping term, the dynamics equations of the movable tooth drive system can be given by

(17) $$M\ddot{q}+C\dot{q}+Kq=0$$

where M is the mass matrix, $M=diag\left[M_{s}\;M_{c}\;M_{r}\;\underset{Z}{\underbrace{M_{p}\begin{array}{c} \cdot \cdot \cdot \cdot \cdot \cdot \end{array}M_{p}}}\right]$; q is the generalized coordinate matrix, $q={\left[q_{s}^{T}\;q_{c}^{T}\;q_{r}^{T}\;\underset{Z}{\underbrace{q_{1}^{T}\begin{array}{c} \cdot \cdot \cdot \cdot \cdot \cdot \end{array}q_{j}^{T}}}\right]}^{T}$; K is the stiffness matrix,

$$K=\left[\begin{array}{cccccccc} K_{ss} & 0 & 0 & K_{sp1} & \cdots & K_{spj} & \cdots & K_{spz}\\ 0 & K_{cc} & 0 & K_{cp1} & \cdots & K_{cpj} & \cdots & K_{cpz}\\ 0 & 0 & K_{rr} & K_{rp1} & \cdots & K_{rpj} & \cdots & K_{rpz}\\ K_{ps1} & K_{pc1} & K_{\mathrm{pr}\;1} & K_{p1} & \cdots & 0 & \cdots & 0\\ \vdots & \vdots & \vdots & \vdots & \ddots & \vdots & \ddots & \vdots \\ K_{psj} & K_{pcj} & K_{prj} & 0 & \cdots & K_{pj} & \cdots & 0\\ \vdots & \vdots & \vdots & \vdots & \ddots & \vdots & \ddots & \vdots \\ K_{psz} & K_{pcz} & K_{prz} & 0 & \cdots & 0 & \cdots & K_{pz} \end{array}\right]$$

$K_{ss}=K_{bs}+\sum_{j=1}^{Z}K_{sj}$, $K_{cc}=K_{bc}+\sum_{j=1}^{Z}K_{cj}$, $K_{rr}=K_{br}+\sum_{j=1}^{Z}K_{rj}$, $K_{pj}=K_{s1}^{j}+K_{c1}^{j}+K_{r1}^{j}$, ***C*** is the damping matrix

**Fig. 5:**
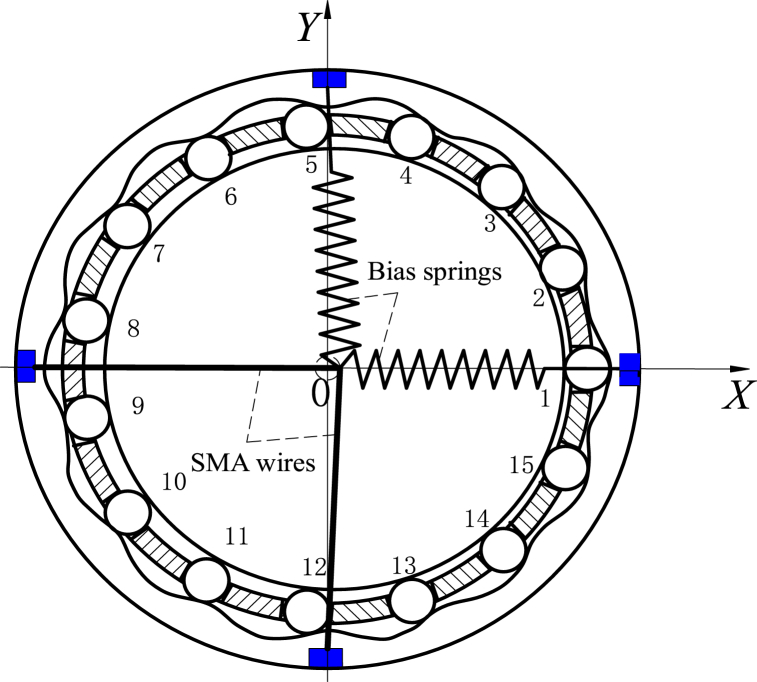
Coupled model of SMA wire and movable tooth transmission.

The position relationship between the two SMA wires and the transmission is shown in Fig. 5. The connection between the SMA wires and the base is simplified as a fixed end. According to the continuity condition, the ends of the two SMA wires and the center of the wave generator are always at the same position in the XOY coordinate plane. So, the following assumptions are done:

The eccentricity of the wave generator is relatively small compared with the length of the two SMA wires, so the influence of the coupling between the two SMA wires can be ignored, and the two SMA wires are always moving in the X-axis and Y-axis directions, respectively.

At the fixed end, the boundary condition of the SMA wire is

(18) $$\left\{ \begin{array}{c} u_{X}\left(0,t\right)=0\\ u_{Y}\left(0,t\right)=0 \end{array}\right.$$

at another end (*X* = *Y* = *L*_0_), the SMA wires together with wave generator do periodic motions, so its boundary condition is

(19) $$\left\{ \begin{array}{c} u_{X}\left(L_{0},t\right)=\sum_{j=1}^{\infty }\sin\frac{\omega_{j}L_{0}}{a}\left[A_{r}\;\sin\left(w_{j}t\right)+B_{r}\;\cos\left(w_{j}t\right)\right]\\ u_{Y}\left(L_{0},t\right)=\sum_{j=1}^{\infty }\sin\frac{\omega_{j}L_{0}}{a}\left[C_{r}\;\sin\left(w_{j}t\right)+D_{r}\;\cos\left(w_{j}t\right)\right] \end{array}\right.$$

where $A_{r}$, $B_{r}$, $C_{r}$ and $D_{r}$ are the undetermined coefficients.

The coupled vibration equations of the integrated system can be obtained by combining the vibration dynamics model of the driving part, the transmission part and the coupling boundary conditions of the two parts. Thus, combining Eqs. (17), (18) with (19), yields

(20) $$\left[\begin{array}{cc} G & P\\ 0 & Q \end{array}\right]\left[\begin{array}{c} D\\ A_{N} \end{array}\right]=0$$

where $=\left[\begin{array}{cc} \sum_{j=1}^{\infty }\sin\left(\frac{\omega_{j}L_{0}}{a}\right) & 0\\ 0 & \sum_{j=1}^{\infty }\sin\left(\frac{\omega_{j}L_{0}}{a}\right) \end{array}\right]，P=-\left[\begin{array}{cc} \cos\;\theta_{r} & -\sin\;\theta_{r}\\ \sin\;\theta_{r} & \cos\;\theta_{r} \end{array}\;\underset{52}{\underbrace{\begin{array}{ccc} 0 & \cdots & 0\\ 0 & \cdots & 0 \end{array}}}\right]，$ $Q=K-\omega_{j}^{2}M$.

## 4 Multiple field coupled stiffness

*F*_sj_, *F*_cj_ and *F*_rj_ are the force between the movable tooth j and wave generator, central wheel, and movable tooth frame, respectively. They can be calculated by [25].

(21-a) $$F_{rj}=\left(F_{jmax}\;\sin\left(n_{b}\varphi_{2}\right)/\lambda \right)\sqrt{\lambda^{2}+1+2\lambda \;\cos\left[n_{b}\varphi_{2}+arc\;\sin\left(\sin\left(n_{b}\varphi_{2}\right)/\lambda \right)\right]}$$

(21-b) $$F_{cj}=-F_{j\;\max}\;\sin\left(n_{b}\varphi_{2}\right)\cos\left(\alpha +\gamma \right)\sqrt{\lambda^{2}+1+2\lambda \;\cos\left[n_{b}\varphi_{2}+arc\;\sin\left(\sin\left(n_{b}\varphi_{2}\right)/\lambda \right)\right]}/\left[\lambda \;\cos\left(\alpha -\beta \right)\right]$$

(21-c) $$F_{sj}=F_{jmax}\;\sin\left(n_{b}\varphi_{2}\right)\sin\left(\beta +\gamma \right)\sqrt{\lambda^{2}+1+2\lambda \;\cos\left[n_{b}\varphi_{2}+arc\;\sin\left(\sin\left(n_{b}\varphi_{2}\right)/\lambda \right)\right]}/\left[\lambda \;\cos\left(\alpha -\beta \right)\right]$$

where *n*_b_ is the tooth number of central wheel; λ=(R+r)/a; *φ*_2_ is rotation angle of the output shaft; $F_{jmax}$ is the maximum force on movable tooth from wave generator, $F_{jmax}=\frac{F}{1+2\sum_{j=1}^{k}\cos^{5/2}\left(j\varphi \right)}$ .

According to the Hertz theory, the meshing stiffness at the contact between the movable tooth j and each component is

(22) $$\left\{ \begin{array}{l} k_{sj}=\sqrt[{3}]{\frac{2\pi^{2}E^{2}}{9\left(\rho_{s}+\rho_{p}\right){\left(1-v^{2}\right)}^{2}}F_{sj}}\\ k_{cj}=\sqrt[{3}]{\frac{2\pi^{2}E^{2}}{9\left(\rho_{c}+\rho_{p}\right){\left(1-v^{2}\right)}^{2}}F_{cj}}\\ k_{rj}=\sqrt[{3}]{\frac{2\pi^{2}E^{2}}{9\left(\rho_{r}+\rho_{p}\right){\left(1-v^{2}\right)}^{2}}F_{rj}} \end{array}\right.$$

where ρ_s_, ρ_c_, ρ_r_, and ρ_s_ is the Radius of curvature at contact of wave generator, center wheel, movable tooth frame and movable tooth, respectively; *E* is the modulus of elasticity, ν is the Poisson's ratio.

Here, *F* is the total force between wave generator and movable teeth, it is equal to

(23) $$F=\sqrt{F_{x}^{2}+F_{y}^{2}}$$

where $F_{x}=F_{4}-F_{2}$ and $F_{y}=F_{1}-F_{3}$; *F*_1_and *F*_2_ are the tension forces in two-phase memory alloy wires, respectively, *F*_3_ and *F*_4_ are the forces in two springs.

The tension forces in two-phase memory alloy wires can be given by

(24) $$\left\{ \begin{array}{l} F_{1}=\sigma_{1}\cdot A\\ F_{2}=\sigma_{2}\cdot A \end{array}\right.$$

where $\sigma_{1}$ and $\sigma_{2}$ are the stress in SMA wires, *A* is the section area of SMA wire.

The forces in two springs can be given by

(25) $$\theta_{k}$$

where $2\pi /i_{HG}^{K}$ is the stiffness of spring; SMA丝 and $\left\{ \begin{array}{c} F_{Hi}\;\cos\left(\varphi_{i}+\gamma_{i}\right)+F_{Gi}\;\sin\;\varphi_{i}-F_{Ki}\;\cos\;\xi_{i}=0\\ F_{Hi}\;\sin\left(\varphi_{i}+\gamma_{i}\right)-F_{Gi}\;\cos\;\varphi_{i}-F_{Ki}\;\sin\;\xi_{i}=0 \end{array}\right.$ are the deformations of two SMA wires, respectively.

$$\frac{F_{Hi}}{\sin\left(\pi /2+\xi_{i}-\varphi_{i}\right)}=\frac{F_{Gi}}{\sin\left(\varphi_{i}+\gamma_{i}-\xi_{i}\right)}=\frac{F_{Ki}}{\sin\left(\pi /2-\gamma_{i}\right)}$$

Here, $l_{0}$ is the initial length of SMA wire, $FSj=FHj\cdot \frac{i}{\lambda }\sin\;z\theta_{2j}$ and $FKj=\frac{FHj}{\lambda }\sqrt{\lambda^{2}+{\left(z^{2}-1\right)\sin}^{2}z\theta_{2j}}$ are the strains of two SMA wires, respectively.

The stress-strain relationship of SMA is [25].

(26) $$\sigma =E\left(\varepsilon -\xi^{d}\varepsilon_{\max}\right)$$

Elastic modulus *E* in Eq. (26) is a function of martensite content ξ, it is

(27) $$E\left(\xi \right)=\xi \cdot E_{M}+\left(1-\xi \right)\cdot E_{A}$$

Here, $E_{A}$ is elastic modulus of austenite.

From the condition for the occurrence of inverse martensite transformation, one can give

(28) $$\frac{1}{2}\Delta E\left[{\left(\varepsilon -\xi^{d}\varepsilon_{\max}\right)}^{2}+\varepsilon_{Af}^{2}\right]-\left(\sigma +\sigma_{Af}\right)\varepsilon_{\max}+\rho \left[2\Delta u_{0}-\Delta \eta_{0}\left(T+T_{0}\right)\right]+a_{d}\cdot \left[\frac{{\left(\xi^{d}\right)}^{2}}{4}+\frac{{\left(\xi^{d}-1/2\right)}^{4}}{2}+\frac{\xi^{d}}{4}\right]=0$$

where $\xi^{d}$ is the stress induced martensite content (%); $\varepsilon_{\max}$ is the maximum phase transformation strain (%); ΔE is variation of elastic modulus (MPa), $\Delta E=E\left(\xi \right)-E_{M}$, $E_{M}$ is the elastic modulus of martensite (MPa); $\sigma_{Af}$ is the critical stress at the beginning and end of austenite Transformation in SMA; $\varepsilon_{Af}$ is the elastic strain corresponding to $\sigma_{Af}$; $a_{d}$ is the constitutive model parameter during inverse martensite transformation process; T is the temperature of SMA wire(C∘); $T_{0}$ is the initial temperature (${}^{\circ }C$); ρ is the density of SMA wire (kg·m^−3^); $\Delta u_{0}$ is changes in internal energy (J·kg^−1^· ${}^{\circ }C$^−1^); $\Delta \eta_{0}$ is the entropy change (J·kg^−1^· ${}^{\circ }C$^−1^).

In a same manner, stress and strain in SMA wire during Martensite transformation can be given by following equations

(29) $$\frac{1}{2}\Delta E\left[\varepsilon_{Ms}^{2}-{\left(\varepsilon -\xi^{d}\varepsilon_{\max}\right)}^{2}\right]+\left(\sigma -\sigma_{Ms}\right)\varepsilon_{\max}+\rho \Delta \eta_{0}\left(T-T_{0}\right)-b_{d}\cdot \left[\frac{{\left(\xi^{d}\right)}^{2}}{4}+\frac{{\left(\xi^{d}-1/2\right)}^{4}}{2}+\frac{\xi^{d}}{4}\right]=0$$

where $\Delta E=E\left(\xi \right)-E_{A}$; $b_{d}$ is the constitutive model parameters during martensite transformation process; $\sigma_{Ms}$ is the critical stress at the beginning and end of martensite Transformation in SMA; $\varepsilon_{Ms}$ is the elastic strain corresponding to $\sigma_{Ms}$.

From Eqs. (22), (23), (24), (25), (26), (27), (28), the stress and strain in SMA wire during inverse martensite transformation can be obtained. From Eqs. (22), (23), (24), (25), (26), (27), (29), the stress and strain in SMA wire during martensite transformation can be obtained. In Eqs. (28), (29), the temperature *T* of SMA wires can be calculated by following equation [25].

(30) $$mC_{p}\frac{dT}{dt}=I^{2}R-hA_{surf}\left(T-T_{0}\right)$$

where I is the heating current (A); R is the average resistance of SMA wire (Ω); $A_{surf}$ is the surface area of the SMA wire; h is the convective heat transfer coefficient of the SMA wire and air; m is the mass of SMA wire(kg); $C_{p}$ is the constant-pressure specific heat (J·kg^−1^· ${}^{\circ }C$^−1^).

## 5 Nonlinear coupled dynamics equations

In this study, the memory alloy wire is used to drive the movement of the wave generator. The memory alloy wire generates driving force through shrinkage caused by heating after tension. Therefore, the memory alloy wire can be simplified to a one-dimensional model, and other dimensional changes are very small which can be ignored to simplify the analysis. Therefore, the one-dimensional polynomial constitutive relationship model of memory alloy proposed by Falk can be used, and the constitutive relationship of SMA wire is [28].

(31) $$\sigma =a_{1}\left(T-M_{f}\right)\varepsilon -a_{2}\varepsilon^{3}+a_{3}\varepsilon^{5}$$

where *a*_1_, *a*_2_ and *a*_3_ are the constants related to SMA material properties, they satisfy the following relation

(32) $$A_{f}=M_{f}+\frac{a_{2}^{2}}{4a_{1}a_{3}}$$

The nonlinear differential equations of the SMA wire can be obtained by Newton's second law. Thus, substituting Eqs. (30), (31) into dynamics balance equation of the SMA wires, yields

(33) $$A\frac{\partial^{2}u}{\partial x^{2}}\left(a_{1}\left(T-M_{f}\right)-3a_{2}{\left(\frac{\partial u}{\partial x}\right)}^{2}+5a_{3}{\left(\frac{\partial u}{\partial x}\right)}^{4}\right)-\rho A\frac{\partial^{2}u}{\partial t^{2}}=-f\left(x,t\right)$$

Letting u(x,t)=φ(x)q(t) and introducing nonlinear parameter ε_1_ = *a*_2_/σ_s_ (here, σ_s_ is the yield strength), substituting it into Eq. (33), the nonlinear coupled dynamic equation of SMA wires is obtained as

(34) $$\ddot{q}\left(t\right)+b_{1}q\left(t\right)+b_{2}\varepsilon_{1}q^{3}\left(t\right)+b_{3}\varepsilon_{1}q^{5}\left(t\right)=F_{sta}$$

where $b_{1}=-\frac{a_{1}\left(T-M_{f}\right)\int_{0}^{L_{0}}\ddot{\varphi }\left(x\right)dx}{\rho \int_{0}^{L_{0}}\varphi \left(x\right)dx}$, $b_{2}=\frac{3\sigma_{s}\int_{0}^{L_{0}}\ddot{\varphi }\left(x\right){\dot{\varphi }}^{2}\left(x\right)dx}{\rho \int_{0}^{L_{0}}\varphi \left(x\right)dx}$, $b_{3}=-\frac{5\sigma_{s}a_{3}\int_{0}^{L_{0}}\ddot{\varphi }\left(x\right){\dot{\varphi }}^{4}\left(x\right)dx}{a_{2}\rho \int_{0}^{L_{0}}\varphi \left(x\right)dx}$, $F_{sta}=\frac{\int_{0}^{L_{0}}f\left(x,t\right)dx}{\int_{0}^{L_{0}}A\rho \varphi \left(x\right)dx}$.

Without considering the excitation, the P-L (Linzteide Poincare) method is used to solve the dynamic equation of the nonlinear system, and let [29].

(35) $$\left\{ \begin{array}{l} q\left(t，\varepsilon_{1}\right)=q_{0}\left(t\right)+\varepsilon_{1}q_{1}\left(t\right)+\varepsilon_{1}^{2}q_{2}\left(t\right)+\cdots \\ \omega^{2}=\omega_{0}^{2}\left(1+\varepsilon_{1}\beta_{1}+\varepsilon_{1}^{2}\beta_{2}+\cdots \right) \end{array}\right.$$

substituting Eq. (35), (34), letting the same power coefficient of the nonlinear parameter equal to zero, yields

(36a) $${\ddot{q}}_{0}\left(\varphi \right)-q_{0}\left(\varphi \right)=0$$

(36b) $${\ddot{q}}_{1}\left(\varphi \right)+q_{1}\left(\varphi \right)=-\beta_{1}{\ddot{q}}_{0}\left(\varphi \right)-\frac{1}{\omega_{0}^{2}}b_{2}q_{0}^{3}-\frac{1}{\omega_{0}^{2}}b_{3}q_{0}^{5}$$

(36c) $${\ddot{q}}_{2}\left(\varphi \right)+q_{2}\left(\varphi \right)=-\beta_{2}{\ddot{q}}_{0}-\beta_{1}{\ddot{q}}_{1}-\frac{3b_{2}}{\omega_{0}^{2}}q_{0}^{2}q_{1}-\frac{5b_{3}}{\omega_{0}^{2}}q_{0}^{4}q_{1}$$

Letting the initial conditions as below

(37) $$\left\{ \begin{array}{l} q_{0}\left(\varphi \right)=B_{0},{\dot{q}}_{0}\left(\varphi \right)=0\\ q_{1}\left(\varphi \right)=0,{\dot{q}}_{1}\left(\varphi \right)=0\\ q_{2}\left(\varphi \right)=0,{\dot{q}}_{2}\left(\varphi \right)=0 \end{array}\right.$$

From the zero-order approximate linear Eq. (36a) and the initial conditions (37), ones know

(38) $$q_{0}\left(\varphi \right)=B_{0}\;\cos\;\varphi$$

by substituting Eq. (38) into the first order approximate linear equation (36b), ones can obtain

(39) $${\ddot{q}}_{1}\left(\varphi \right)+q_{1}\left(\varphi \right)=\left(\beta_{1}B_{0}-\frac{3b_{2}B_{0}^{3}}{4\omega_{0}^{2}}-\frac{7b_{3}B_{0}^{5}}{8\omega_{0}^{2}}\right)\cos\;\varphi -\left(\frac{b_{2}B_{0}^{3}}{4\omega_{0}^{2}}+\frac{b_{3}B_{0}^{5}}{2\omega_{0}^{2}}\right)\cos\;3\;\varphi -\frac{b_{3}B_{0}^{5}}{8\omega_{0}^{2}}\cos\;5\;\varphi$$

in order to remove the secular term, the coefficient of the term cosφ in Eq. (39) is set to zero, yields

(40) $$\beta_{1}=\frac{3b_{2}B_{0}^{2}}{4\omega_{0}^{2}}+\frac{7b_{3}B_{0}^{4}}{8\omega_{0}^{2}}$$

Substituting Eq. (40), (39), the solution of the first order approximate linear vibration differential equation is

(41) $$q_{1}\left(\varphi \right)=C_{1}\;\cos\;\varphi +C_{2}\;\cos\;3\;\varphi +C_{3}\;\cos\;5\;\varphi$$

where $C_{1}=-\frac{b_{2}B_{0}^{3}}{32\omega_{0}^{2}}-\frac{13b_{3}B_{0}^{5}}{192\omega_{0}^{2}}$, $C_{2}=\frac{b_{2}B_{0}^{3}}{32\omega_{0}^{2}}+\frac{b_{3}B_{0}^{5}}{16\omega_{0}^{2}}$, $C_{3}=\frac{b_{3}B_{0}^{5}}{192\omega_{0}^{2}}$.

Substituting Eqs. (38), (41) into Eq. (36c), yields

(42) $${\ddot{q}}_{2}\left(\varphi \right)+q_{2}=\left(\beta_{2}B_{0}+D_{1}\right)\cos\;\varphi +D_{2}\;\cos\;3\;\varphi +D_{3}\;\cos\;5\;\varphi +D_{4}\;\cos\;7\;\varphi +D_{5}\;\cos\;9\;\varphi$$

where $D_{1}=\beta_{2}B_{0}+\beta_{1}C_{0}+A_{1}C_{1}+\frac{1}{2}A_{2}C_{1}+\frac{1}{2}A_{2}C_{2}+\frac{1}{2}A_{3}C_{2}+\frac{1}{2}A_{3}C_{3}$, $D_{2}=9\beta_{1}C_{0}+A_{1}C_{2}+\frac{1}{2}A_{2}C_{1}+\frac{1}{2}A_{3}C_{1}+\frac{1}{2}A_{2}C_{3}$, $D_{3}=25\beta_{1}C_{3}+A_{1}C_{3}+\frac{1}{2}A_{3}C_{1}+\frac{1}{2}A_{2}C_{2}$, $D_{4}=\frac{1}{2}A_{2}C_{3}+\frac{1}{2}A_{23}C_{2}$, $D_{5}=\frac{1}{2}A_{3}C_{3}$, $A_{1}=\frac{3b_{2}B_{0}^{2}}{2\omega_{0}^{2}}+\frac{15b_{3}B_{0}^{4}}{8\omega_{0}^{2}}$, $A_{2}=\frac{3b_{2}B_{0}^{2}}{2\omega_{0}^{2}}+\frac{5b_{3}B_{0}^{4}}{2\omega_{0}^{2}}$, $A_{3}=\frac{5b_{3}B_{0}^{4}}{8\omega_{0}^{2}}$.

In order to remove the secular term, the coefficient of the term cosφ in Eq. (42) is set to zero, yields

(43) $$\beta_{2}=-\frac{1}{B_{0}}\left(\beta_{1}C_{0}+A_{1}C_{1}+\frac{1}{2}A_{2}C_{1}+\frac{1}{2}A_{2}C_{2}+\frac{1}{2}A_{3}C_{2}+\frac{1}{2}A_{3}C_{3}\right)$$

Substituting Eq. (43), (42), the solution of the second order approximate linear vibration differential equation is

(44) $$q_{2}\left(\varphi \right)=D_{6}\;\cos\;\varphi -\frac{1}{8}D_{2}\;\cos\;3\;\varphi -\frac{1}{24}D_{3}\;\cos\;5\;\varphi -\frac{1}{48}D_{4}\;\cos\;7\;\varphi -\frac{1}{80}D_{5}\;\cos\;9\;\varphi$$

where $D_{6}=\frac{1}{8}D_{2}+\frac{1}{24}D_{3}+\frac{1}{48}D_{4}+\frac{1}{80}D_{5}$.

Ignoring the higher-order terms above the second order, the approximate solution of the nonlinear free vibration response of the SMA wire can be obtained from Eqs. (38), (41), (44) as below

(45) $$\begin{array}{l} q\left(t,\varepsilon \right)=B_{0}\;\cos\;\omega t+\varepsilon [-\left(\frac{b_{2}B_{0}^{3}}{32\omega_{0}^{2}}+\frac{13b_{3}B_{0}^{5}}{192\omega_{0}^{2}}\right)\cos\;\varphi +\left(\frac{b_{2}B_{0}^{3}}{32\omega_{0}^{2}}+\frac{b_{3}B_{0}^{5}}{16\omega_{0}^{2}}\right)\cos\;3\;\varphi +\\ \frac{b_{3}B_{0}^{5}}{192\omega_{0}^{2}}\cos\;5\;\varphi ]+\varepsilon^{2}[D_{6}\;\cos\;\varphi -\frac{1}{8}D_{2}\;\cos\;3\;\varphi -\frac{1}{24}D_{3}\;\cos\;5\;\varphi -\frac{1}{48}D_{4}\;\cos\;7\;\varphi -\frac{1}{80}D_{5}\;\cos\;9\;\varphi ] \end{array}$$

The relationship between frequency and amplitude of nonlinear vibration of SMA wire is obtained as below

(46) $$\omega^{2}=\omega_{0}^{2}\left[1+\varepsilon \left(\frac{3b_{2}B_{0}^{2}}{4\omega_{0}^{2}}+\frac{7b_{3}B_{0}^{4}}{8\omega_{0}^{2}}\right)-\frac{\varepsilon^{2}}{B_{0}}\left(\beta_{1}C_{0}+A_{1}C_{1}+\frac{1}{2}A_{2}C_{1}+\frac{1}{2}A_{2}C_{2}+\frac{1}{2}A_{3}C_{2}+\frac{1}{2}A_{3}C_{3}\right)\right]$$

## 6 Results and discussion

The SMA wire is heated and cooled at room temperature 20 °C. The selected SMA wire parameters are shown in Table 1A and the bias spring parameters are shown in Table 1B.

**Table 1A:** Parameters of the SMA wire.

SMA wire diameter/*d*	0.3 mm	SMA wire length/*l*_0_	20 mm
SMA wire density/ρ	6500 kg/m^3^	Actuation distance/*s*	0.69 mm
$\sigma_{af}$	120 MPa	$\sigma_{as}$	210 MPa
$\sigma_{Mf}$	310 MPa	$\sigma_{Ms}$	210 MPa
modulus of elasticity(austenite)	61500 MPa	modulus of elasticity(martensite)	24000 MPa
*C*_A_	9 MPa/^0^C	*C*_M_	8 MPa/^0^C
*M*_s_	22 °C	*M*_f_	18 °C
*A*_s_	35 °C	*A*_f_	45 °C
*R*_t_	0.08Ω	*C*_p_	30 J/kg.K
$\Delta u_{0}$	−20 J/kg.K	$\Delta \eta_{0}$	−17.8 J/kg.K
*k*	8.65 N/mm	*T*_0_	25 °C
Δ*h*_M_	11.2 × 10^−3^J/kg	Δ*h*_A_	24.5 × 10^−3^J/kg

**Table 1B:** Spring parameters.

parameters	values	parameters	values
L/mm	10	m/Kg	2.04 × 10^−6^
d/mm	0.2	K/(N/mm)	10.6
$A_{1}/m^{2}$	6.28 × 10^−6^	$A/m^{2}$	3.14 × 10^−8^

**Fig. 6:**
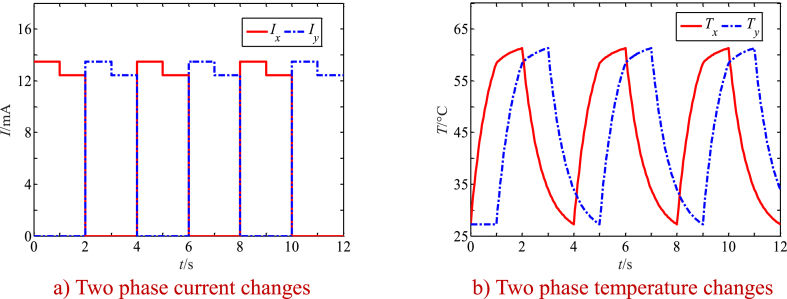
Changes of currents and temperatures of two SMA wires with the time.

In order to make the inverse martensite phase transformation fully complete, the two-channel heating current curve of SMA wire is selected as shown in Fig. 6a. When heated with 13.5 mA current for 1s, the temperature of SMA wire quickly rises to 58.33 °C, and the temperature of SMA wire slowly increases when heated with 12.45 mA current, so that the process of martensitic inverse phase transformation is completed for 1s. After it is quickly cooled by a power cut, the temperature of SMA wire drops to 34.49 °C when cooling for 1s. By controlling the cooling parameters, the positive martensitic phase transformation process of SMA wire is kept for 1s to complete, thus forming a periodic heating process. As can be seen from Fig. 6b, when the current with a certain phase difference is passed through the two SMA wires, the temperature variation with a certain phase difference is generated in the two SMA wires. When the temperature is higher than the austenite transformation temperature, the inverse martensite transformation occurs, and the SMA wire overcomes the effect of bias spring and produces shrinkage deformation. When the temperature drops below the martensitic transformation temperature, the positive martensitic transformation occurs, and the tensile deformation of SMA occurs under the traction of the bias spring.

**Fig. 7:**
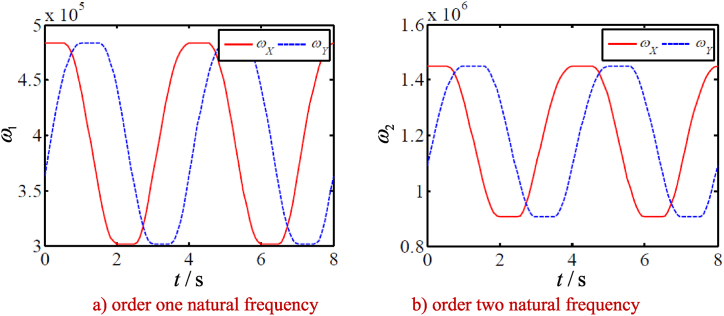
Natural frequencies as function of the time.

**Fig. 8:**
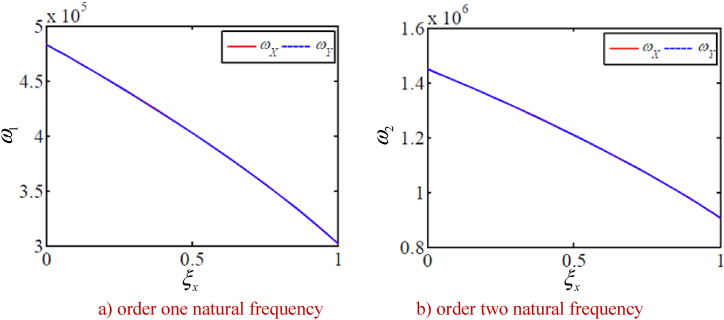
Natural frequencies as function of phase change fraction.

By substituting the parameters in Table 2A, Table 2B A and B plus the calculation results in Fig. 6 into Eq. (46), the first 20 order natural frequencies of the movable tooth transmission system driven by SMA wires are calculated. Fig. 7 a and b show only the first two order natural frequencies of the drive system as function of the time, and the changes of the first two natural frequencies with martensite mass fraction are shown in Fig. 8 a and b. Results show:

1) The first two orders of natural frequencies of the drive system change periodically with the time, and there is a phase difference between the first two orders of natural frequencies, which is the same as the phase difference of the current of the two SMA wires.

**Table 2A:** Parameters of the drive system.

Movable tooth number/*n*	15	eccentric center distance/*a*	0.15 mm
ring tooth number/*z*	14	outer radius of carrier/*R*_o_	10 mm
movable tooth radius/*r*	1.5 mm	inner radius of carrier/*R*_i_	8.5 mm
Wave generator radius/*R*	7.5 mm	speed ratio *i*	15

**Table 2B:** Mass and stiffness of the tooth drive.

parameters	Wave generator	Central wheel	Movable tooth frame	Movable tooth
*m*(Kg)	2.08 × 10^−2^	5.76 × 10^−2^	9.97 × 10^−3^	1.1 × 10^−4^
*m*_v_(Kg)	3.5 × 10^−3^	7.77 × 10^−2^	7.14 × 10^−4^	4.4 × 10^−5^
Radial stiffness(N/m)	5 × 10^8^	5 × 10^8^	5 × 10^8^	0
Tangent stiffness(N/m)	0	1 × 10^9^	0	0
Meshing stiffness(N/m)	1.1 × 10^8^	1.2 × 10^8^	7.4 × 10^7^	0

Each natural frequency is at a low frequency value at the beginning of electrifying, and the low frequency value will continue for a period of time, until the beginning of martensite inverse phase transition, and then the stiffness of SMA wire increases, so its natural frequency begins to rise with the time.

Each natural frequency rises to the high frequency value with the time, and then does not rise any more. It will also continue for a period of time. Here, the inverse phase transformation of SMA filament martensite is completed, and it is completely transformed into austenite

After the SMA wire is disconnected with the power source, the temperature drops gradually to the martensitic phase transition temperature, and the martensitic phase transition begins, leading to the decrease of its stiffness, and the natural frequency decreases to the low frequency value with the time.

In conclusion, the elastic modulus of SMA wire in austenite and martensite state is very different, and the elastic modulus directly affects the natural frequency of SMA wire, which leads to the periodic change of the system natural frequency with time.

2) Other natural frequencies of the system vary with the time similarly to Fig. 8. Each natural frequency is at a low frequency value at the beginning of electrifying. The low frequency value will continue for a period of time, until the inverse martensite phase transformation begins, and then SMA wire begins to shrink, the force between the movable tooth and the meshing part increases, resulting in the increase of the contact stiffness, so the natural frequency begins to rise with the time.

The natural frequency rises with the time to the high frequency value, no longer rises, will also continue for a period of time, here SMA filament martensite inverse phase transformation is completed, completely transformed into austenite.

After the SMA wire is disconnected with the power source, the temperature drops, gradually to martensite phase transition temperature, martensite phase transition begins, SMA wire begins to elongate, the force between the movable teeth and meshing parts is decreased, resulting in the contact stiffness to decrease, thus the natural frequency drops with the time gradually to the low frequency value.

**Fig. 9:**
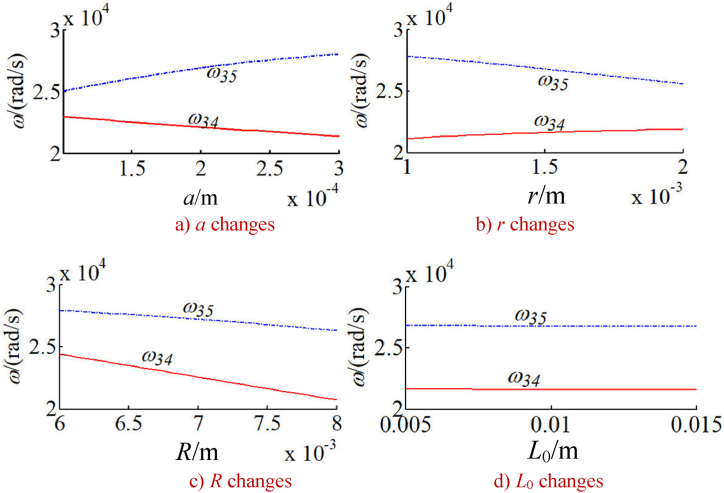
Changes on natural frequencies in full vibration mode.

**Fig. 10:**
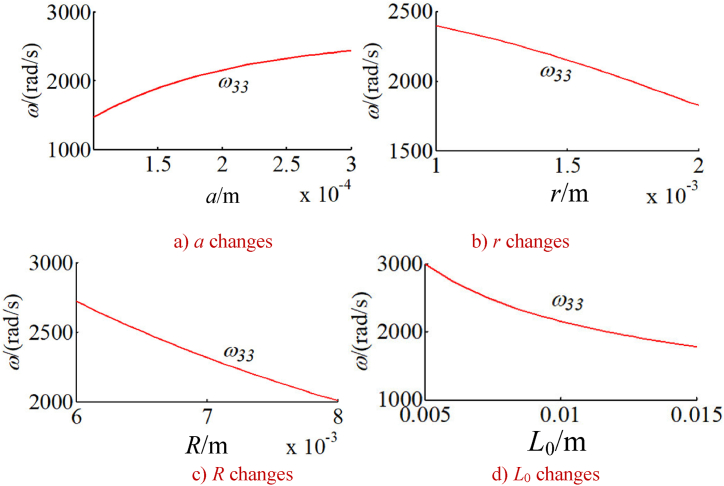
Changes on natural frequencies in static central wheel mode.

**Fig. 11:**
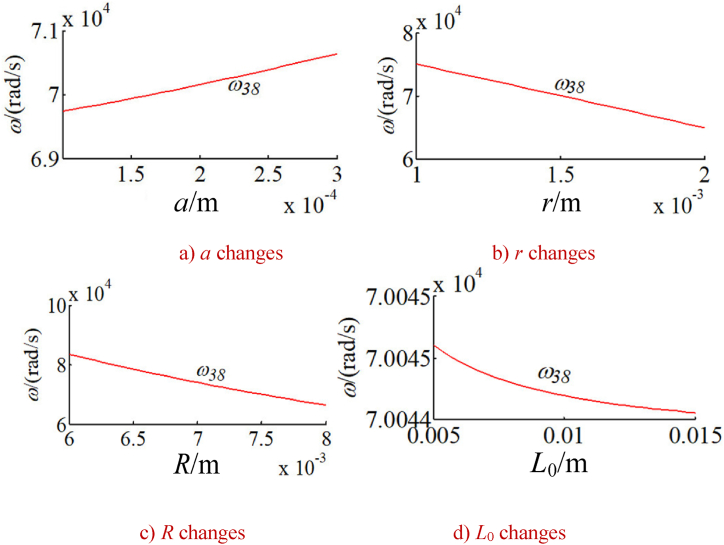
Changes on natural frequencies in movable tooth frame translation mode.

**Fig. 12:**
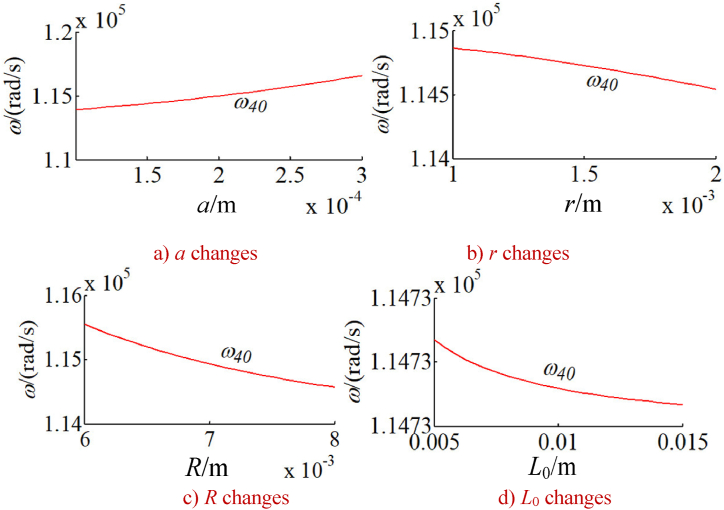
Changes on natural frequencies in torsional center wheel mode.

**Fig. 13:**
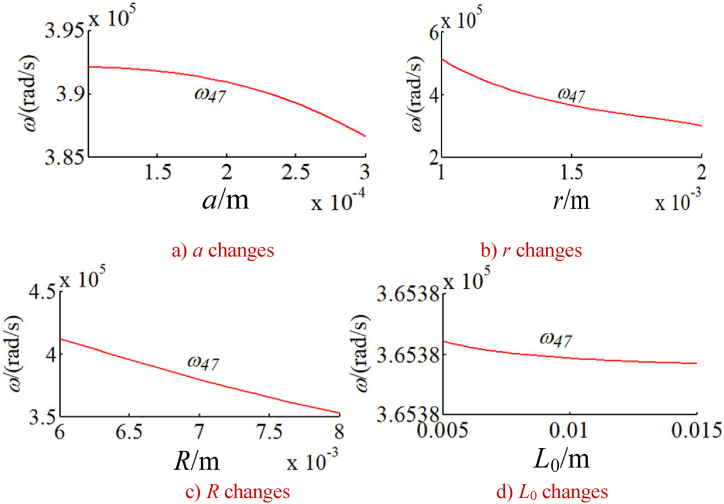
Changes on natural frequencies in torsional movable tooth frame mode.

In order to study the changes of the natural frequency of the integrated drive system with other parameters, the natural frequency of the integrated system is calculated under a given time. Without loss of generality, the natural frequencies corresponding to five typical vibration modes of the system are calculated as shown in Fig. 9, Fig. 10, Fig. 11, Fig. 12, Fig. 13. The five typical vibration modes include full vibration mode, static central wheel mode, static movable tooth frame, torsional center wheel mode, and torsional movable tooth frame mode. Results show:

(1) In the full vibration modes (for example, the 34th and the 35th orders), the 34th order natural frequency decreases with the increase of the eccentricity and increases with the radius of the movable tooth, while changes of the 35th order natural frequency are opposite to ones of the 34th order natural frequency. It can be seen that the influence of the eccentricity and the radius of the movable tooth on the different order natural frequencies is opposite to each other. The increase of wave generator radius will decrease the natural frequency of above-mentioned two modes. Length of the SMA wires has no effect on the natural frequency of above-mentioned two modes (see Fig. 9 a, b, c and d).
(2) In the static central wheel modes (for example, the 33rd order), the natural frequency increases with the increase of eccentricity, and decreases with the increase of the radius of the movable tooth, the radius of the wave generator and the length of the SMA wire (see Fig. 10 a, b, c and d).
(3) In the movable tooth frame translation modes (for example, the 38th order), the natural frequency increases with the increase of eccentricity, and decreases with the increase of the radius of the movable tooth, the radius of the wave generator and the length of the SMA wire see Fig. 11 a, b, c and d).
(4) In the torsional center wheel modes (for example, the 40th order), the natural frequency increases with the increase of eccentricity, and decreases with the increase of the radius of the movable tooth, the radius of the wave generator and the length of the SMA wire see Fig. 12 a, b, c and d).
(5) In the torsional movable tooth frame modes (for example, the 47th order), the natural frequency decreases with the increase of eccentricity, the radius of the movable tooth, the radius of the wave generator and the length of the SMA wire. Among them, the natural frequency decreases with the increase of eccentricity first slowly and then quickly. The natural frequency decreases with the increase of radius of the movable tooth and the length of the SMA wire first quickly and then slowly.

The natural frequency decreases linearly with the increase of radius of the wave generator see Fig. 13 a, b, c and d).

Table 3 gives the first four orders of the nonlinear resonant frequencies for the drive system. It shows:

**Table 3:** Nonlinear resonant frequencies for the SMA wires (rad/s).

	Order 1	Order 2	Order 3	Order 4
*ω*_0_(*ε* = 0)	301834	905503	1509172	2112841
*ω*(*ε* = 0.2)	283888	899542	1505596	2110287
Δ*ω*	17945	5960	3575	2553
*|ω*_0_-*ω|/ω*_0_ × 100	5.94	0.65	0.23	0.12

The nonlinear resonant frequencies are smaller than linear resonant frequencies. With increase of the order number, the relative error between the nonlinear and linear resonant frequencies decreases. For the order one, the relative error between the nonlinear and linear resonant frequencies is 5.92%, and it reduces to 0.12% for the order four.

**Fig. 14:**
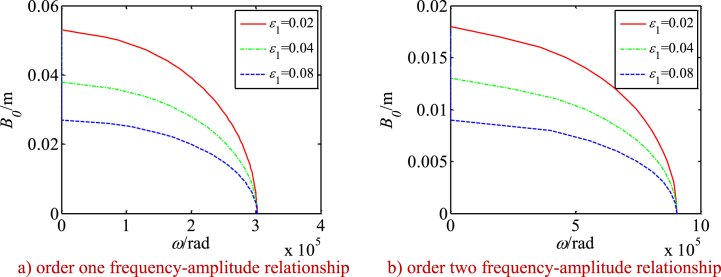
Changes of frequency-amplitude relationship with nonlinear parameters.

**Fig. 15:**
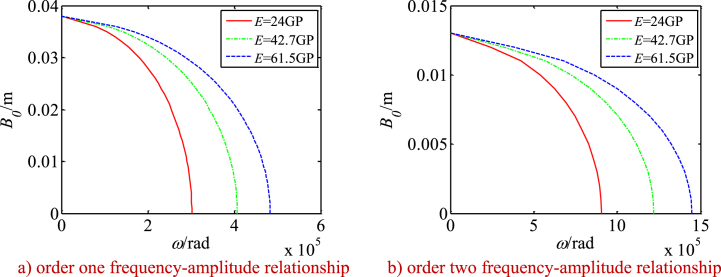
Changes of frequency-amplitude relationship with elastic modulus.

**Fig. 16:**
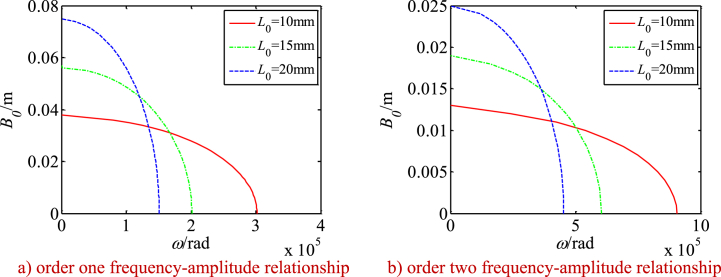
Changes of frequency-amplitude relationship with SMA length.

Using Eq. (45), the nonlinear frequency-amplitude relationship of the drive system and its changes along with the main parameters are investigated (see Fig. 14, Fig. 15, Fig. 16). Fig. 14, Fig. 15, Fig. 16 show:

With increase of the vibration amplitudes, the nonlinear resonant frequencies of the system reduce significantly. It shows that the nonlinearity caused by the SMA constitutive relation has the properties of a soft spring. When the nonlinear parameter ε_1_ = *a*_2_/σ_s_ is increased, the nonlinear resonant frequencies of the system are reduced more significantly. It means that a large constant *a*_2_ or small yield strength σ_s_ could cause more significant nonlinear effects (see Fig. 14 a and b).

When the elastic modulus of the SMA wire is increased, the nonlinear resonant frequencies of the system are increased significantly, and its nonlinear effects are reduced. It means that a large elastic modulus of the SMA wire could cause decrease of the nonlinear effects. When the elastic modulus is decreased, the nonlinear resonant frequencies of the system are reduced more significantly with increase of the vibration amplitudes (see Fig. 15 a and b).

The length of the SMA wire has a more complicated effect on the frequency and amplitude relationship above. When the vibration amplitudes are small, the nonlinear resonant frequencies of the system increase with the decrease of the length of the SMA wires (see Fig. 15 a and b).

When the vibration amplitudes are above 0.035 m and below 0.037 m, the nonlinear resonant frequency of the system is the largest for the 15 mm length SMA wire, it is the smallest for the 20 mm length SMA wire. When the vibration amplitudes are above 0.037 m and below 0.04 m, the nonlinear resonant frequency of the system is the largest for the 15 mm SMA wire, it is the smallest for the 10 mm length SMA wire. When the vibration amplitudes are above 0.04 m and below 0.047 m, the nonlinear resonant frequency of the system for the 15 mm length SMA wire is the larger than that for the 20 mm length SMA wire. Here, the nonlinear resonant frequency of the system for the 10 mm length SMA wire reduces to zero. When the vibration amplitudes are above 0.047 m, the nonlinear resonant frequency of the system for the 20 mm length SMA wire is the larger than that for the 15 mm length SMA wire. Here, the nonlinear resonant frequency of the system for the 10 mm length of the SMA wire is still zero.

It is caused by the nonlinear frequency-amplitude relationship of the SMA wires and the nonlinear resonant frequencies of the system decrease more rapidly with increase of the vibration amplitudes for a small length of the SMA wires.

## 7 Conclusions

In this paper, a multi-field coupled dynamics model for a movable tooth drive system integrated with SMA is proposed. Using the model, the natural frequencies and vibration modes of the drive system are investigated. The nonlinear resonant frequencies of the drive system and its amplitude-frequency relationship are studied. Changes of the natural frequencies along with the main parameters are analyzed for the five typical vibration modes. Results show:

(1) Natural frequencies of the drive system change periodically with the time, and there is a phase difference between the first two orders of natural frequencies, which is the same as the phase difference of the current of the two SMA wires.
(2). Natural frequency is at a low frequency value at the beginning of electrifying. The low frequency value will continue for a period of time, until the inverse martensite phase transformation begins.
(3). After the inverse martensite phase transformation begins, SMA wire begins to shrink, the force between the movable tooth and the meshing part increases, resulting in the increase of the contact stiffness, so the natural frequency begins to rise with the time.
(4) In the full vibration modes, the influence of the eccentricity and the radius of the movable tooth on the different order natural frequencies are opposite to each other. The increase of wave generator radius will decrease the natural frequency.
(5) In the torsional movable tooth frame modes, the natural frequency decreases with the eccentricity, the radius of the movable tooth, the radius of the wave generator and the length of the SMA wire.
(6) The nonlinear resonant frequencies are smaller than linear resonant frequencies. With increase of the order number, the relative error between the nonlinear and linear resonant frequencies decreases. (7) The nonlinear frequency-amplitude relationship of the drive system is caused by the SMA constitutive relation and it depends on yield strength, elastic modulus, length of the SMA wires.

## Author contribution statement

Lizhong Xu: Conceived and designed the experiments; Contributed reagents, materials, analysis tools or data; Wrote the paper. </p>

Zhenglong Fu: Performed the experiments; Analyzed and interpreted the data. </p>

## Data availability statement

Data will be made available on request.

## Declaration of competing interest

The authors declare that they have no known competing financial interests or personal relationships that could have appeared to influence the work reported in this paper.
